# Identification and Biochemical Characterization of the Serine Biosynthetic Enzyme 3-Phosphoglycerate Dehydrogenase in *Marchantia polymorpha*

**DOI:** 10.3389/fpls.2018.00956

**Published:** 2018-07-16

**Authors:** Hiromichi Akashi, Eiji Okamura, Ryuichi Nishihama, Takayuki Kohchi, Masami Y. Hirai

**Affiliations:** ^1^RIKEN Center for Sustainable Resource Science, Yokohama, Japan; ^2^Graduate School of Bioagricultural Sciences, Nagoya University, Nagoya, Japan; ^3^Graduate School of Biostudies, Kyoto University, Kyoto, Japan

**Keywords:** activation, amino acids, inhibition, liverwort, phosphate, phosphorylated pathway, serine

## Abstract

L-serine is an important molecule in all living organisms, and thus its biosynthesis is considered to be regulated according to demand. 3-Phosphoglycerate dehydrogenase (PGDH), the first committed enzyme of the phosphorylated pathway of L-serine biosynthesis, is regulated by negative feedback from L-serine in bacteria. In the case of the vascular plant *Arabidopsis thaliana*, two PGDH isozymes out of three are inhibited by L-serine and activated by L-alanine, L-valine, L-methionine, L-homoserine, and L-homocysteine, suggesting a more complicated regulatory mechanism of L-serine biosynthesis in *A. thaliana* than in bacteria. However, it remains to be clarified whether the activation mechanism of PGDH by amino acids is conserved in land plants. In this study, we identified the sole isozyme of PGDH in the liverwort *Marchantia polymorpha* (MpPGDH) and elucidated its biochemical characteristics. Mp*PGDH* cDNA encodes a 65.6 kDa protein that contains a putative transit peptide for chloroplast localization. MpPGDH shares 75–80% identity with *A. thaliana* isozymes and forms a homotetramer *in vitro*. Recombinant MpPGDH exhibited an optimal pH of 9.0, apparent Michaelis constants of 0.49 ± 0.04 and 0.096 ± 0.010 mM for 3-PGA and NAD^+^, respectively, and apparent maximum velocity of 5.65 ± 0.10 μmol⋅min^−1^⋅mg^−1^, similar to those of *A. thaliana* isozymes. Phosphate ions were found to stabilize MpPGDH, suggesting that phosphate ions are also a crucial factor in the regulation of serine biosynthesis via the phosphorylated pathway in *Marchantia polymorpha*. MpPGDH was inhibited by L-serine in a cooperative manner and was activated by L-alanine, L-valine, L-methionine, L-homoserine, and L-homocysteine to a lesser extent than it is in *A. thaliana*. The results suggest that an ancestral PGDH of land plants was inhibited byL-serine and slightly activated by five other amino acids.

## Introduction

L-serine plays pivotal roles not only as a building block of proteins but also as a precursor of various important biomolecules such as amino acids, nucleic acid bases, phospholipids, and sphingolipids. In plants, L-serine also plays an important role in the response to environmental stresses such as high salinity, flooding, and low temperature (Ho and Saito, 2001). L-Serine is synthesized in plants by three pathways: the glycolate pathway, the glycerate pathway, and the phosphorylated pathway (Ros et al., 2014; Igamberdiev and Kleczkowski, 2018). The plant-specific glycolate pathway is a part of photorespiration and is considered to be important in photosynthetic organs (Kleczkowski and Givan, 1988; Bauwe et al., 2010; Maurino and Peterhansel, 2010). On the other hand, the phosphorylated pathway, which is conserved among plants, animals, and bacteria, is considered to play a role in non-photosynthetic organs or under conditions when the photorespiration does not work (Ros et al., 2013, 2014).

The phosphorylated pathway consists of three sequential reactions catalyzed by 3-phosphoglycerate dehydrogenase (PGDH), 3-phosphoserine aminotransferase (PSAT), and 3-phosphoserine phosphatase (PSP). The precursor 3-phosphoglycerate (3-PGA) is first reversibly oxidized by PGDH, utilizing NAD^+^ as a cofactor to form 3-phosphohydroxypyruvate (3-PHP). The second enzyme PSAT converts 3-PHP to 3-phosphoserine (3-PS) using L-glutamate as an amino donor. The last step is dephosphorylation of 3-PS to form L-serine in a reaction catalyzed by PSP. The first committed enzyme PGDH is allosterically inhibited by L-serine in bacteria (Sugimoto and Pizer, 1968; Schuller et al., 1995; Thompson et al., 2005; Dey et al., 2008). PGDH of *Mycobacterium tuberculosis* (MtPGDH) is comprised of three domains: a catalytic domain, an aspartate kinase–chorismate mutase–TyrA (ACT) domain, and an allosteric substrate binding (ASB) domain (Grant, 2012). MtPGDH is cooperatively inhibited by L-serine, which binds to the ACT domain in the presence of anions such as phosphate and chloride, while it is non-cooperatively inhibited by L-serine in the absence of these anions (Dey et al., 2005; Xu and Grant, 2014). The quaternary structure of MtPGDH varies among homodimers, homotetramers, and homooctamers, depending on phosphate concentration, which leads to changes in catalytic activity and sensitivity to inhibition of L-serine (Xu and Grant, 2014). Therefore, phosphate ions, as well as L-serine, are key molecules for regulation of MtPGDH enzyme activity.

*Arabidopsis thaliana* possesses three PGDH isozymes, which share 75–81% identity with each other and 35–37% identity with MtPGDH. AtPGDH1 (At4g34200) and AtPGDH3 (At3g19480) are inhibited by L-serine, while AtPGDH2 (At1g17745) is not (Benstein et al., 2013; Okamura and Hirai, 2017). The expression of genes encoding the three AtPGDH isozymes shows different organ specificity. *AtPDGH1* is expressed in the tips of cotyledons, shoot and root apical meristems, the vasculature of leaves and roots, and at points of lateral root emergence (Benstein et al., 2013; Toujani et al., 2013). By contrast, *AtPGDH2* is expressed in the vasculature of shoot and shoot apical meristems, but its expression is more pronounced in the vasculature of the root. *AtPGDH3* is expressed strongly in the cotyledons and weakly in the leaves, but its expression is completely absent from the roots and meristematic tissue in 10-day-old seedlings (Benstein et al., 2013; Toujani et al., 2013). Furthermore, three PGDH isozymes play different physiological functions. Knockout of the *AtPGDH1* gene leads to developmental defects in embryos, male gametophytes, and roots (Pagnussat et al., 2005; Benstein et al., 2013; Toujani et al., 2013). Silencing of the *AtPGDH1* gene leads to visible lesion phenotypes under high CO_2_ conditions (Benstein et al., 2013), indicating that AtPGDH1 plays a role in L-serine supply under high CO_2_ conditions where photorespiration is repressed, and thus L-serine biosynthesis via the glycolate pathway is repressed. *AtPGDH1* is co-expressed with the genes involved in biosynthesis of tryptophan and tryptophan-derived indole glucosinolates under regulation of the transcriptional factors MYB34 and MYB51. Silencing of the *AtPGDH1* gene also leads to a decrease in tryptophan-derived auxin and indole glucosinolates (Benstein et al., 2013), indicating a specific function of AtPGDH1 in the supply of L-serine used as the precursor of tryptophan biosynthesis. Therefore, regulation of PGDH activity is crucial for managing these physiological events.

Recently, we found that AtPGDH1 and AtPGDH3 are not only inhibited by L-serine but are activated by L-alanine, L-valine, L-methionine, L-homoserine, and L-homocysteine in a cooperative manner, whereas AtPGDH2 is not (Okamura and Hirai, 2017). This led to questions regarding whether the PGDHs of other land plants are activated by these amino acids, and if so, when in the course of evolution land plants acquired the activation mechanism. Because liverworts belong to a basal lineage of land plants, an understanding of the regulation mechanism of PGDH in liverwort species will lead to better understanding of evolution of PGDH enzymes in land plants.

In this study, we identified the single-copy PGDH gene in the liverwort *Marchantia polymorpha* and characterized the basic biochemical properties of its encoded protein. We demonstrated that the PGDH of *Marchantia polymorpha* is not only inhibited by L-serine but is also activated by other five amino acids.

## Materials and Methods

### Homology Search for Serine Biosynthetic Genes and Multiple Alignments

The homologs of *AtPGDH1* (At4g34200), *AtPSAT1* (At4g35630), and *AtPSP* (At1g18640) in *Physcomitrella patens* were searched using blastp. Then, the top hit sequence of respective genes (PHYPADRAFT_122014, PHYPADRAFT_129784, and PHYPADRAFT_13475) was used as a query for a tblastn search against the transcriptome database of *Marchantia polymorpha* in MarpolBase^1^ (Bowman et al., 2017) to identify Mapoly0030s0029 (MpPGDH), Mapoly0033s0118 (MpPSAT), and Mapoly0023s0019 (MpPSP). These were used as queries for BLAST search against the genome database of *Marchantia polymorpha*. Amino acid sequences of PGDHs from *A. thaliana* (AtPGDHs), *Mycobacterium tuberculosis* (MtPGDH), and *Marchantia polymorpha* (MpPGDH) were aligned using MUSCLE with MEGA7 software (Edgar, 2004; Kumar et al., 2016). Alignments were rendered using ESPript^2^ (Gouet et al., 1999; Robert and Gouet, 2014).

### Reagents

Nicotine amide adenine dinucleotide (NAD) disodium salt, D-(−)-3-phosphoglyceric acid (3-PGA) disodium salt, and molecular weight marker for size exclusion chromatography analysis were purchased from Sigma-Aldrich Co., Ltd. (St. Louis, MO, United States). Molecular weight markers for sodium dodecyl sulfate-polyacrylamide gel electrophoresis (SDS-PAGE) analysis were purchased from BioDynamics Laboratory Inc. (Tokyo, Japan). The salt mixture of Gamborg’s B5 medium and Good’s buffers were purchased from Wako Pure Chemical Industries, Ltd. (Osaka, Japan).

### Plant Materials and Growth Conditions

Male accession of *Marchantia polymorpha*, Takaragaike-1 (Tak-1), was cultured aseptically on half-strength Gamborg’s B5 agar medium containing 1% agar at 22°C under 50–60 μmol m^−2^ s^−1^ white light (16 h light/8 h dark photoperiod). Three-week-old thalli grown from the gemma were harvested, immediately frozen with liquid nitrogen, and used for RNA extraction.

### RNA Preparation and Amplification of Mp*PGDH* cDNA

Total RNA was extracted from the thalli using the QIAGEN RNeasy Plant Mini Kit (Qiagen, Germantown, MD, United States). The first strand of cDNA was synthesized using the SuperScript III First-Strand Synthesis System for RT-PCR (Thermo Fisher Scientific) and primer MpPGDH-N (5′-CACCATGGCGGCGACGAGTGCCGTAGCAGCGGTG-3′). Then, cDNAs were amplified using the Platinum PCR SuperMix High Fidelity (Thermo Fisher Scientific) and the primer sets of MpPGDH-N and MpPGDH-C (5′-TTACAACTTAAGGAAGACAAACTCTTCGAT-3′). PCR amplification was carried out as follows: pre-denature, 3 min at 95°C; denature, 95°C for 30 s; annealing, 55°C for 30 s; extension, 72°C for 1 min, 25 cycles; final extension, 72°C for 5 min. The PCR products were analyzed by agarose gel electrophoresis and purified using a QIAquick Gel Extraction Kit (Qiagen). The cDNA was directly inserted into pENTR-D-TOPO (Thermo Fisher Scientific) and then sequenced to obtain pENTR-MpPGDH.

### Construction of Vectors

The protein expression vector for MpPGDH without the transit peptide was constructed as previously reported (Okamura and Hirai, 2017). Briefly, PCR was performed using PrimeSTAR Max DNA Polymerase (Takara Bio Inc., Kyoto, Japan) with pENTR-MpPGDH as a template and the primer sets of MpPGDH-*Spe*I (5′-AAAGCTTTGACTAGTATTGTCGGCAAGCCCACCGT-3′) and MpPGDH-*Not*I (5′-GGCTTATGCGGCCGCTTACAACTTAAGGAAGACAAACTCTTCGAT-3′). Then, the amplified fragment was assembled with the *Spe*I-*Not*I site on pPAL7 (Bio-RAD) by an In-Fusion HD Cloning Kit (Takara) to obtain pPAL7-MpPGDH. The genetic complementation vector was constructed using lac inducible pTV118N (Takara). PCR was performed by the same method as construction of the protein expression vector pPAL7-PGDH-MP, except that the primers MpPGDH-*Nco*I-F (5′-CACACAGGAAACAGACCATGATTGTCGGCAAGCCCACCGTCCTCG-3′) and MpPGDH-*Kpn*I-R (5′-CTAGAGGATCCCCGGTTACAACTTAAGGAAGACAAACTCT-3′) were used. The amplified fragment was assembled into the *Nco*I-*Kpn*I site on pTV118N to obtain pTV118N-MpPGDH.

### Functional Complementation of *Escherichia coli* Serine Auxotroph Mutant

The L-serine-auxotroph *E. coli* JW 2880 strain (TG1Δ*serA*::KmFRT), in which the PGDH coding gene (*serA*) was disrupted by insertion of the kanamycin resistance gene (Baba et al., 2006), was transformed with pTV118N-MpPGDH. The transformants were grown on Luria–Bertani (LB) medium containing 50 μg/mL kanamycin and 100 μg/mL carbenicillin. Then, the transformants were streaked on M9 media containing 25 μg/mL kanamycin and 50 μg/mL carbenicillin under induction of *lac* promotor and incubated for 10 days at 37°C. As a control, the transformants were grown on M9 media containing 0.2 mM L-serine.

### Protein Expression and Purification

Protein expression in *E. coli* and purification were performed as follows. The transformant of *E. coli* BL21 CodonPlus (DE3)-RIL (Agilent) carrying pPAL7-MpPGDH was pre-incubated at 20°C for 1 h in liquid LB medium supplemented with 100 μg/mL ampicillin and 50 μg/mL chloramphenicol, and then incubated for induction of protein expression for 48 h at 20°C after adding isopropyl-β-D-thiogalactopyranoside (final concentration of 0.5 mM). Then, cells were harvested by centrifugation at 9,000 × *g* for 15 min at 4°C and stored at −30°C. Tag-free recombinant proteins were prepared using Profinity eXact Purification Resin (Bio-RAD) for affinity purification. Briefly, cell pellets were suspended in 300 mM sodium phosphate buffer (pH 9.0) and sonicated on ice for 10 min. Crude extracts were then centrifuged for 15 min at 9,000 × *g* and the supernatant was filtered using Millex-HP Syringe Filter Unit (0.45 μM, 33 mm, Merck-Millipore). The filtered supernatants were applied to a Profinity eXact Purification Resin and on-column incubations were performed for 30 min at 20°C to eliminate the affinity tag eXact Fusion-Tag from the MpPGDH recombinant proteins. Eluted fractions were then concentrated and desalted with 300 mM sodium phosphate buffer by ultrafiltration using an Amicon Ultra-4 Centrifugal filter Unit (MWCO 10,000; Merck-Millipore).

### Size Exclusion Chromatography

The molecular mass of MpPGDH in its native state was estimated by size exclusion chromatography analysis in the presence of 100 mM phosphate using AKTA Start instruments with HiPrep 16/60 Sephacryl S-300 HR columns (GE Healthcare) at a flow rate of 0.5 mL/min. The molecular mass was calculated based on standard curves of thyroglobulin (669 kDa), apoferritin (443 kDa), β-amylase (200 kDa), albumin (66 kDa), and carbonic anhydrase (29 kDa) in 100 mM sodium phosphate buffer (pH 9.0).

### Spectrophotometric Assays of Recombinant MpPGDH and Calculation of Kinetic Parameters

3-PGA oxidation activity was assayed as previously described (Okamura and Hirai, 2017). Briefly, the activity was assayed in 600 μL reaction mixtures containing 0.1 M Good’s buffer (described below), 1 mM dithiothreitol, 10 mM 3-PGA, 1 mM NAD^+^, 0.1 M NaCl, and 2.0 μg of recombinant enzyme. Reactions were initiated by adding 3-PGA (Saski and Pizer, 1975; Dey et al., 2005). 3-PGA oxidation activities were determined according to increases in the absorbance of NADH (340 nm) using a UV-2700 spectrophotometer (Shimadzu, Kyoto, Japan). Optimal pH values for 3-PGA oxidation activity were determined in the presence of 10 mM 3-PGA and 1 mM NAD^+^ at pH intervals of 0.5 between 6.0 and 11.0. Good’s buffers were used as follows: MES-NaOH for pH 6.0 and 6.5; HEPES-NaOH for pH 7.0, 7.5, and 8.0; TAPS-NaOH for pH 8.5 and 9.0; CHES-NaOH for pH 9.5 and 10.0, and CAPS-NaOH for pH 10.5 and 11.0. Data were collected from three technical replicates. To determine kinetic parameters for 3-PGA oxidation activity at pH 9.0, 3-PGA was added at 0.1, 0.2, 0.5, 0.7, 1, 2, 5, 7, and 10 mM with a fixed NAD^+^ concentration of 1 mM. In separate experiments, NAD^+^ was applied at 0.01, 0.02, 0.05, 0.07, 0.1, 0.2, 0.5, 0.7, and 1.0 mM with a fixed 3-PGA concentration of 10 mM. Initial velocities were determined from the slopes of plots of NADH formation versus incubation time. The molar extinction coefficient (𝜀) of NADH at 340 nm was 6.2 × 10^3^. Kinetic parameters [apparent Michaelis constants (*K*_m_^app^) and apparent maximum velocities (*V*_max_^app^)] were calculated by fitting specific activities (*v*) to Michaelis–Menten equations (Michaelis and Menten, 1913; Michaelis et al., 2011). Data fitting to the Michaelis–Menten equation was performed using the Enzyme kinetics module in SigmaPlot (Systat Software, San Jose, CA, United States).

The sensitivity of MpPGDH enzyme activity to L-serine, L-alanine, L-valine, L-methionine, L-homoserine, and L-homocysteine was tested using the aforementioned method with slight modifications. Specific activities were determined at 10 mM 3-PGA and 1 mM NAD^+^ in the presence of 0, 0.1, 0.2, 0.5, 0.7, 1, 2, 5, 7, 10, 20, and 50 mM of L-alanine, L-valine, L-methionine, and L-homoserine, respectively, and 0, 0.001, 0.002, 0.005, 0.007, 0.01, 0.02, 0.05, 0.07, 0.1, 0.2, and 0.5 mM L-homocysteine, respectively.

The effects of phosphate ions on the stability of MpPGDH were determined as follows. The purified MpPGDH enzymes were incubated in 0.33 M sodium phosphate buffer (pH 9.0) or 30 mM sodium phosphate buffer (pH 9.0) with or without 0.3 M sodium chloride for 24 h at 25°C. Next, PGDH activity was assayed by the aforementioned method for determination of 3-PGA oxidation activity.

## Results

### Identification of the Phosphorylated Pathway Genes in *Marchantia polymorpha*

We examined whether *Marchantia polymorpha* possesses genes encoding the enzymes of the phosphorylated pathway. BLAST searches against the *Marchantia polymorpha* transcriptome and genome databases identified respective single-copy genes encoding PGDH (Mapoly0030s0029), PSAT (Mapoly0033s0118), and PSP (Mapoly0023s0019). According to the guidelines for gene nomenclature in *Marchantia* (Bowman et al., 2016), we designated the genes as Mp*PGDH*, Mp*PSAT*, and Mp*PSP*, respectively.

**FIGURE 1:**
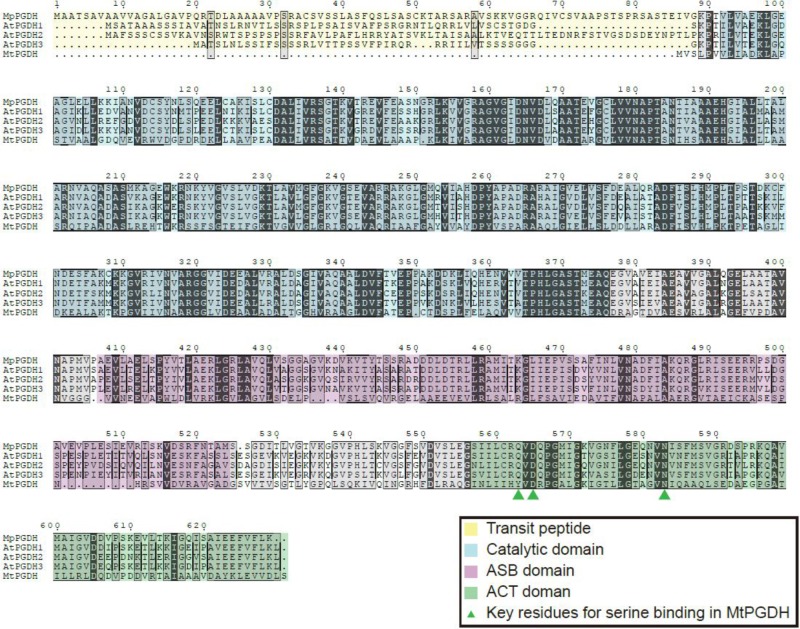
Multiple amino acid sequence alignments of PGDHs. Amino acid sequences of PGDHs from *Marchantia polymorpha* (MpPGDH), *A. thaliana* (AtPGDH1 to 3), and *Mycobacterium tuberculosis* (MtPGDH) are shown with predicted transit peptides (yellow), catalytic domains (blue), ASB domains (violet), and ACT domains (green). Green triangles represent the key L-serine binding residues in MtPGDH.

The deduced amino acid sequence of Mp*PGDH* showed 80, 75, 79, and 39% identity to AtPGDH1, AtPGDH2, AtPGDH3, and MtPGDH, respectively (**Figure 1**). The deduced amino acid sequences of Mp*PSAT* and Mp*PSP* shared 67 and 57% identity to AtPSAT1 and AtPSP, respectively (Supplementary Figure S1).

### Mp*PGDH* Encodes a Functional PGDH

*In silico* expression analysis using MarpolBase^3^ revealed that Mp*PGDH* is expressed in the thallus, sporeling, archegoniophore, antheridiophore, and sporophyte (Supplementary Figure S2). Then, we cloned Mp*PGDH* cDNA from the thallus of a male line, Tak-1. The cDNA encodes 65.6 kDa protein containing a putative transit peptide for chloroplast localization, the catalytic domain, the ASB domain, and the ACT domain (**Figure 1**).

**FIGURE 2:**
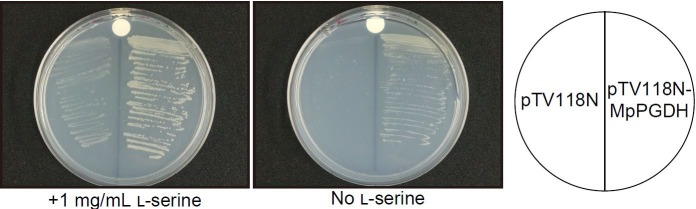
Complementation of an *E. coli* PGDH-defective mutant with Mp*PGDH* cDNA. *E. coli* strain JW2880 (TG1Δ*serA*::KmFRT) was transformed with the expression vector pTV118N-MpPGDH carrying Mp*PGDH* cDNA or with the empty vector pTV118N as a negative control. The transformed bacteria were cultured on M9 minimal medium agar plates with (left) or without (right) 1 mg mL^−1^ of L-serine.

To examine whether the cDNA actually encodes a functional PGDH, cDNA without the transit peptide was expressed in a serine auxotrophic *PGDH*-deficient mutant of *E. coli* (Ho et al., 1999; Baba et al., 2006). The transformants grew on the medium without Ser supplementation (**Figure 2**), indicating that the cDNA encodes a functional PGDH enzyme.

### Basic Biochemical Properties of MpPGDH

**FIGURE 3:**
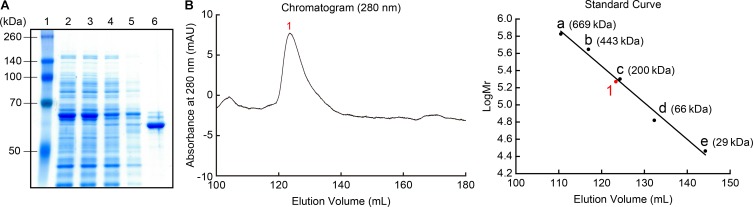
Recombinant MpPGDH protein. **(A)** Purification of recombinant MpPGDH. A total *E. coli* lysate and the sample at each purification step were electrophoresed on SDS-PAGE gel and stained with Coomassie Brilliant Blue. Lane 1, molecular weight marker; Lane 2, crude lysate; Lane 3, supernatant; Lane 4, flow through; Lane 5, wash fraction; Lane 6, elute fraction. **(B)** Size exclusion chromatography of the recombinant MpPGDH. The left panel shows the chromatogram from the size exclusion chromatography analysis. The right panel shows the standard curve for the estimation of molecular mass. 1, MpPGDH; a, thyroglobulin; b, apoferritin; c, β-amylase; d, albumin; e, carbonic anhydrase.

The recombinant MpPGDH protein without the transit peptide was expressed in *E. coli* and purified for further biochemical analysis. SDS-PAGE analysis indicated that the size of the recombinant protein was approximately 60 kDa, which was consistent with the theoretical molecular mass of MpPGDH (57.7 kDa) (**Figure 3A**). Because MtPGDH comes in multiple forms and the forms are related to sensitivity to inhibitor L-serine (Xu and Grant, 2014), we performed size exclusion chromatography to investigate the quaternary structure of MpPGDH (**Figure 3B**). The chromatogram indicated that MpPGDH formed a homotetramer *in vitro*.

**FIGURE 4:**
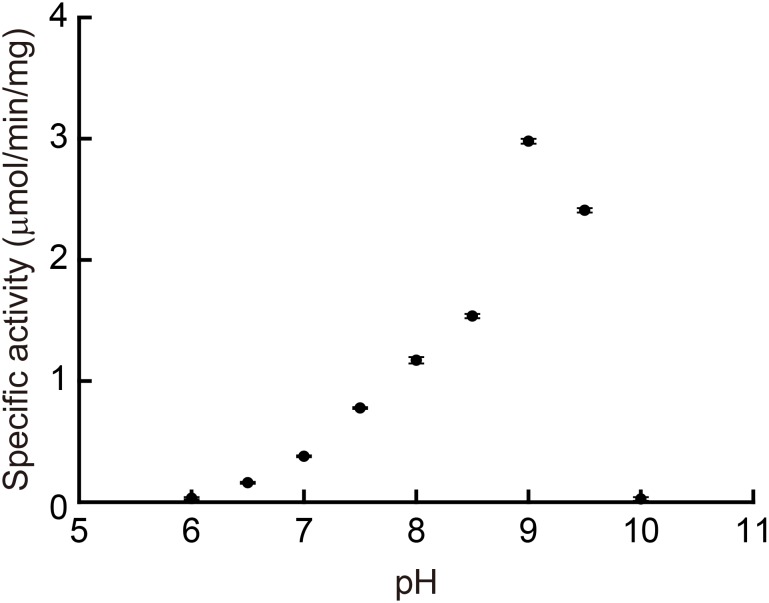
Optimal pH of MpPGDH. The specific activities of MpPGDH are shown at various pH values. Data are presented as means and standard errors of three technical replicates.

We determined the enzymatic properties of the recombinant MpPGDH *in vitro*. **Figure 4** indicates that the optimal pH of the MpPGDH activity was 9.0. This value was similar to those of AtPGDH1 (pH 9.0), AtPGDH2 (pH 10.0), and AtPGDH3 (pH 9.5) (Okamura and Hirai, 2017).

**FIGURE 5:**
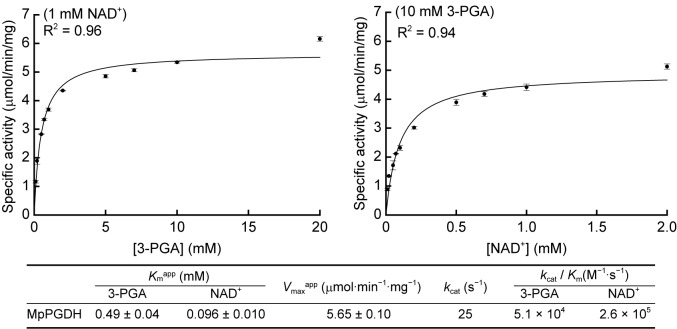
Kinetic parameters of MpPGDH. Michaelis–Menten plots for calculating apparent Michaelis constants (*K*_m_^app^) and apparent maximum velocities (*V*_max_^app^) are shown for 3-PGA (left) and NAD^+^ (right). Data are presented as means and standard errors of three technical replicates.

Then we determined the apparent Michaelis constants (*K*_m_^app^) and apparent maximum velocities (*V*_max_^app^) of MpPGDH for 3-PGA and NAD^+^ at pH 9.0 (**Figure 5**). The *K*_m_^app^ for 3-PGA and NAD^+^ was 0.49 ± 0.04 and 0.096 ± 0.010 mM, respectively, whereas the *V*_max_^app^ was 5.65 ± 0.10 μmol⋅min^−1^⋅mg^−1^. The values were within the same order as those of AtPGDHs (Okamura and Hirai, 2017).

### Effect of Phosphate Ions on the Stability of MpPGDH

**FIGURE 6:**
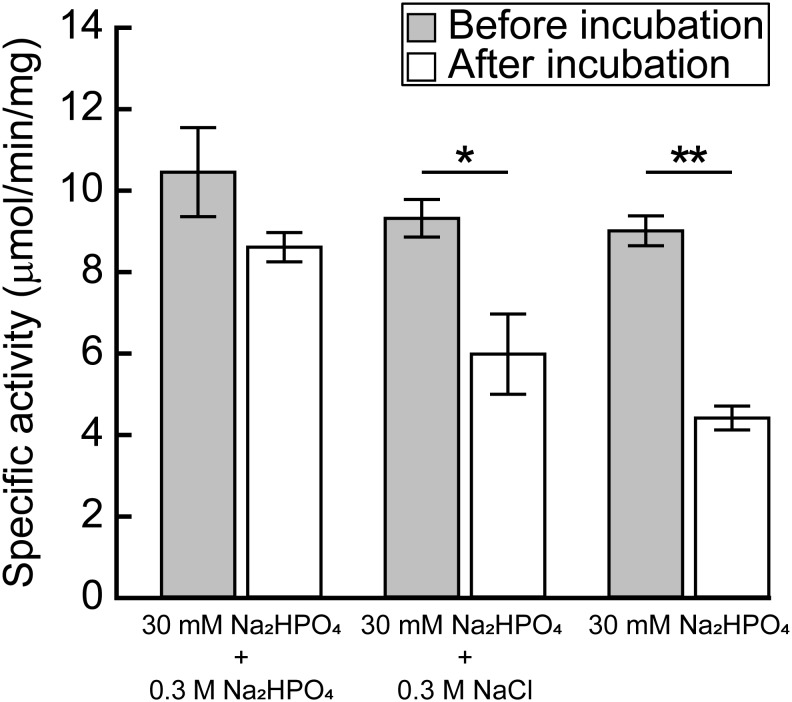
Effect of ionic strength on the stability of MpPGDH. Freshly prepared MpPGDH was incubated in 30 mM Na_2_HPO_4_ + 0.3 M Na_2_HPO_4_, 30 mM Na_2_HPO_4_ + 0.3 M NaCl, or 30 mM Na_2_HPO_4_ for 24 h and immediately used for enzyme assay. Data are presented as means and standard deviations of triplicates using three separately purified recombinant enzymes (*n* = 3). Asterisks indicate significant differences (^∗∗^*p* < 0.001, ^∗^*p* < 0.05, Welch’s *t*-test).

Dey et al. (2005) reported that the activity of the recombinant MtPGDH was almost lost within 24 h after purification when it was stored in 20 mM phosphate buffer, but not in 100 mM phosphate buffer or in 20 mM phosphate buffer containing 100 mM sodium chloride or potassium chloride. The results indicated that ionic strength originating from phosphate and chloride ions is crucial for the stability of purified MtPGDH enzyme (Dey et al., 2005). We examined the stability of purified recombinant MpPGDH in the presence of phosphate and chloride ions. After 24-h incubation in 0.33 M sodium phosphate (the buffer used in protein purification) at its optimal pH 9.0, MpPGDH retained 82% enzyme activity (**Figure 6**). In contrast, MpPGDH enzymes incubated in 0.03 M sodium phosphate with or without 0.3 M NaCl retained 55 and 49% enzyme activity, respectively (**Figure 6**). The results indicate that the stability of MpPGDH is not only dependent on ionic strength, but also on some specific nature of phosphate. This is in contrast to the case of MtPGDH, where 100 mM phosphate and 100 mM chloride stabilized MtPGDH enzyme to a similar extent (Dey et al., 2005).

### MpPGDH Activity Is Cooperatively Inhibited by L-Serine

**FIGURE 7:**
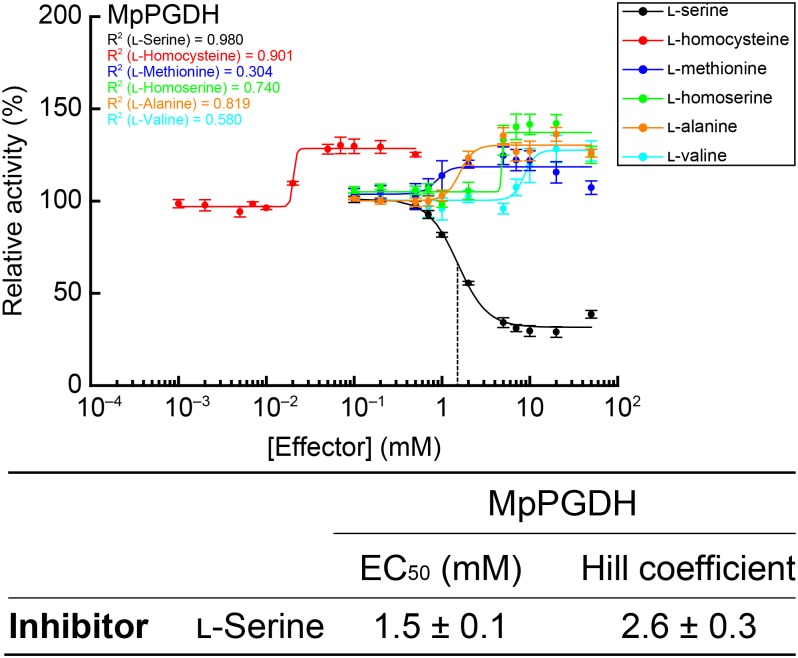
Dose responses of MpPGDH activities to amino acids. Specific activities of MpPGDH at various concentrations of amino acids were measured and are expressed as relative activity to those in the absence of amino acids. Data are presented as means and standard errors of two technical replicates by using two separately purified recombinant enzymes (*n* = 4). Regression curves to the Hill equation are shown with determination coefficients (*R*^2^). Half-maximal effective concentration (EC_50_) and Hill coefficient of L-serine is shown in the table.

Enzyme activity of AtPGDH1 and AtPGDH3 from *A. thaliana* is cooperatively inhibited by L-serine and activated by L-alanine, L-valine, L-methionine, L-homoserine, and L-homocysteine, while AtPGDH2 is insensitive to these amino acids (Okamura and Hirai, 2017). In this study, we examined whether these six amino acids affect MpPGDH activity. **Figure 7** indicates that MpPGDH activity was inhibited to 40% by L-serine in a cooperative manner. The EC_50_ value was 1.5 ± 0.1 mM, which was comparable to that of AtPGDH3 (1.3 ± 0.1 mM) and within the same range as that of AtPGDH1 (6.6 ± 0.3 mM). Moreover, MpPGDH was activated to 135% by L-alanine (at >5 mM), 128% by L-valine (>20 mM), 122% by L-methionine (>7 mM), 140% by L-homoserine (>7 mM), and 130% by L-homocysteine (>0.05 mM). This result was consistent with the previous finding that homotetrameric AtPGDHs are sensitive to amino acids (Okamura and Hirai, 2017). The plots did not fit to the Hill equation (*R*^2^ < 0.91), and thus did not support cooperativity of activation. Considering the biological concentrations of these amino acids in plants, these results suggest that MpPGDH is mainly regulated by cooperative inhibition by L-serine *in vivo*.

## Discussion

### Possible Molecular Mechanism of PGDH Stabilization by Phosphate Ions

In this study, we demonstrated that MpPGDH is stabilized by phosphate ions at its optimal pH of 9.0. The mechanism of stabilization is attributable not only to the ionic strength owing to phosphate ions, but also to some characteristics of phosphate ions such as their chemical structure, because chloride ion did not stabilize MpPGDH to the same extent as phosphate ions at the same concentration (**Figure 6**). Xu and Grant (2014) demonstrated that the ASB domain of MtPGDH functions as a phosphate ion binding site. In our study, the amino acid sequence alignment (**Figure 1**) suggested that MpPGDH possesses the ASB domain. Therefore, binding of phosphate ion at the ASB domain may be a crucial biochemical property of MpPGDH involved in regulation of its enzyme activity *in vivo*. The concentration of phosphate ions in chloroplast stroma was estimated to be 20–35 mM (Dietz and Heber, 1984), and it might dramatically change depending on phosphate ion availability in the environment, light/dark conditions, and phosphate-producing biochemical reactions in chloroplasts, affecting the stability of MpPGDH enzymes.

### Possible Molecular Evolution of PGDH Associated With Activation Mechanisms

This study demonstrated that MpPGDH activity is cooperatively inhibited by L-serine to 40%. The extent of inhibition was comparable to those in AtPGDH1 and AtPGDH3 (Okamura and Hirai, 2017). By contrast, L-alanine, L-valine, L-methionine, L-homoserine, and L-homocysteine activated MpPGDH, although cooperativity was not observed (**Figure 7**). The extent of activation was somewhat smaller than those in AtPGDH1 and AtPGDH3 (140–200%; Okamura and Hirai, 2017). As was the case with AtPGDH1 and AtPGDH3, activation by L-homocysteine was observed at the lowest concentration among the activator amino acids (**Figure 7**). Based on these results, acquisition of the activation mechanism would at least predate the time land plants emerged.

In the case of MtPGDH, its inhibitor L-serine binds to the ACT domain at the Y461, D463, and N481 residues (Dey et al., 2008). The multiple alignment indicates that the corresponding aspartate (D) and asparagine (N) residues are conserved in amino acid-sensitive MpPGDH, AtPGDH1, and AtPGDH3, as well as in amino acid-insensitive AtPGDH2 (**Figure 1**). This indicates that the existence of these residues is not sufficient for the regulation of PGDH activity. Instead, a homotetrameric structure seems necessary for regulation (**Figure 3**; Okamura and Hirai, 2017). During the course of land plant evolution, some features of the PGDH structure necessary for the regulation might have been lost in the process of gene duplication and subfunctionalization to produce amino acid-insensitive isozymes.

### Future Studies on Serine Metabolism

In addition to the specific regulatory mechanism of PGDH activity, regulation of the entire serine metabolism is an important process that needs to be clarified. The phosphorylated pathway was induced by faster serine turnover during photorespiration, which was caused by elevated serine:glyoxylate aminotransferase activity (Modde et al., 2017). In contrast, phosphohydroxypyruvate, a product of PGDH, inhibited cytosolic hydroxypyruvate reductase (HPR-2) activity (Kleczkowski et al., 1990), whose reverse reaction is thought to be involved in serine biosynthesis via the glycerate pathway (Igamberdiev and Kleczkowski, 2018). Such inter-pathway regulations among serine biosynthetic pathways may be closely related to a balancing of carbon, nitrogen, and sulfur metabolisms. A database search of MarpolBase indicated that the *Marchantia polymorpha* genome contains the putative genes for the glycolate pathway of serine biosynthesis. Since *Marchantia polymorpha* is in a critical phylogenetic position in the evolution of land plants and genetic research tools and resources have been established (Ishizaki et al., 2016), *Marchantia polymorpha* can be a good experimental system to examine the regulation of serine metabolism.

## Author Contributions

MYH conceived the research. EO and MYH designed the research. HA and EO conducted the wet experiments and analyzed the data. RN and TK conducted BLAST search. HA, EO, and MYH wrote the manuscript.

## Conflict of Interest Statement

The authors declare that the research was conducted in the absence of any commercial or financial relationships that could be construed as a potential conflict of interest.

## Footnotes

1 http://marchantia.info/

2 http://espript.ibcp.fr

3 http://marchantia.info/
