# Supplementary material for: Identification and Biochemical Characterization of the Serine Biosynthetic Enzyme 3-Phosphoglycerate Dehydrogenase in Marchantia polymorpha
Source: Front Plant Sci. 2018 Jul 16;9:956. doi: 10.3389/fpls.2018.00956 (PMC6054995; doi:10.3389/fpls.2018.00956)
Supplement: Supplementary file 1 [file Presentation_1.PDF]

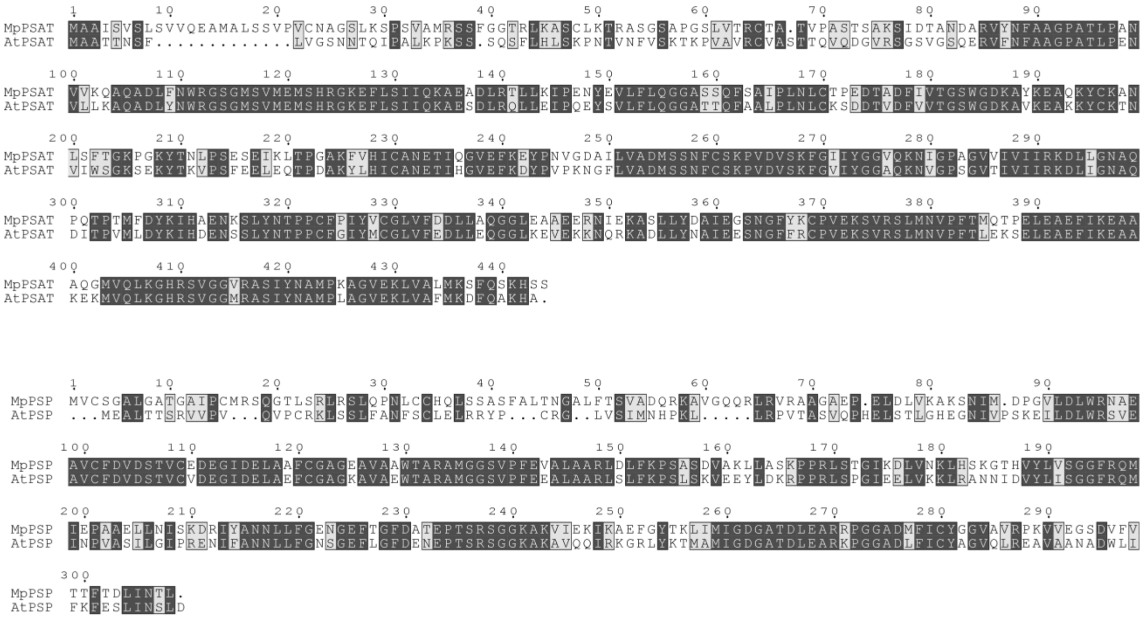

Supplementary Figure 1. Multiple amino acid sequence alignments of PSAT and PSP. Amino acid sequences of PSAT and PSP from *A. thaliana* (AtPSAT1 and AtPSP) and *M. polymorpha* (MpPSAT and MpPSP) were aligned.

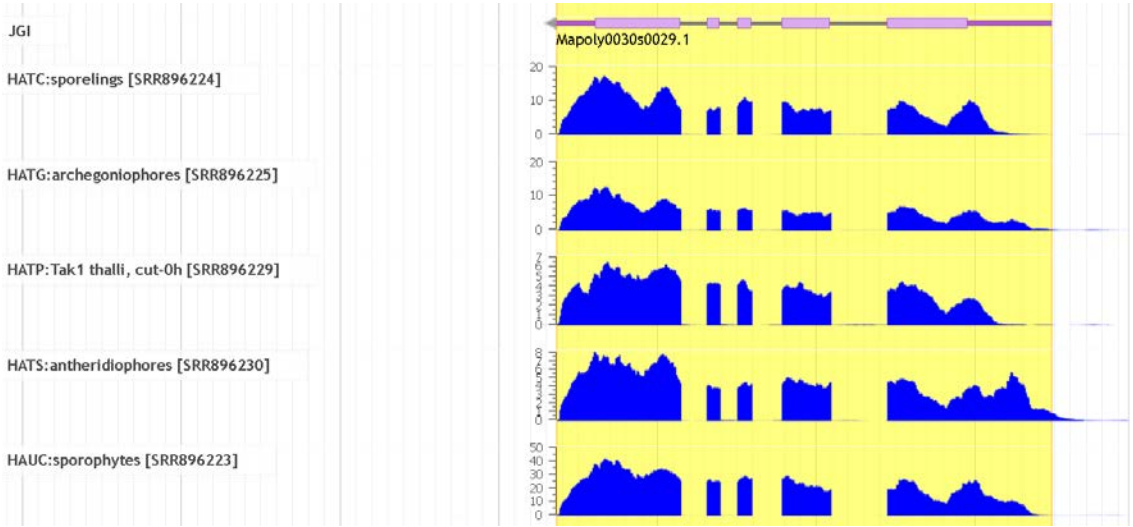

Supplementary Figure 2. *In silico* expression analysis of Mp*PGDH*.

The expression levels of Mp*PGDH* (Mapoly0030s0029.1) are graphically indicated using MarpolBase (http://marchantia.info/).
